# Small amphipathic peptides are responsible for the assembly of cruciferin nanoparticles

**DOI:** 10.1038/s41598-017-07908-z

**Published:** 2017-08-10

**Authors:** Hui Hong, Ali Akbari, Jianping Wu

**Affiliations:** Department of Agricultural, Food and Nutritional Science, University of Alberta, Edmonton, Alberta T6G 2P5 Canada

## Abstract

Amphipathic peptides are versatile building blocks for fabricating well-ordered nanostructures, which have gained much attention owing to their enormous design possibilities and bio-functionalities. However, using amphipathic peptides from natural proteins to create tunable nanostructures is challenging because of their heterogeneity and great tendency to form aggregates. Here we fabricated two well-defined nanoparticles from cruciferin amphipathic peptides by integrating top-down and bottom-up approach. Alkali hydrolysis (pH 12, 120 °C for 30 min) was introduced to break down intact cruciferin into peptides (top–down). The cruciferin peptides and their fractions were then assembled into nanoparticles (bottom–up) in the presence of calcium ions. The permeate fraction from 10 kDa cut-off membrane formed smaller nanoparticles (F1-NPs) (around 82 nm) than that of unfractionated cruciferin peptides (CRU-NPs, around 185 nm); the electrostatic and hydrophobic interactions were the main driving forces for particle formation. LC-MS/MS analysis characterised that the small amphipathic peptides (X_n1_Z_n2_X_n3_Z_n4_, n_1–4_ = 0~5), composed of alternating hydrophobic (X) and hydrophilic (Z) amino acid with a length of 5–15 and 5–20 residues for F1-NPs and CRU-NPs, respectively, were responsible for particle formation. Our study established the mechanism of particle formation of the cold gelation is through assembly of amphipathic peptides.

## Introduction

Polymeric nanocarriers are extensively explored as vehicles for encapsulation and delivery of bioactive compounds and therapeutic molecules^1–3^. Synthetic peptides have emerged in recent years as building blocks for polymeric nanocarrier fabrication because of their well-defined and fine-tuned properties^4–6^. However, increasing concerns on synthetic nanocarrier including potential toxicity, biocompatibility, immunogenicity, and high cost have renewed the interest in developing natural alternatives. Although food proteins are criticized for their possible allergenicity, heterogeneity, and aggregation, as naturally-derived polymers, food proteins are gaining popularity because they are cost-effective, renewable, GRAS (generally regarded as safe)^7^, and easily available. Food proteins can be prepared as building blocks for nanocarrier fabrication under conditions without using toxic chemicals or organic solvents that synthetic route usually involved^8^. Therefore, a range of food proteins, such as soy protein^9^, whey protein^10^, zein^11^, gelatin^12^, and albumin^13^, have been used for nanocarrier fabrication through a variety of methods including electrospinning^9^, glycation^10^, antisolvent coprecipitation^11^, miniemulsion^12^, and PRINT process^13^.

Canola, one of the major global oilseed crops, accounted for approximately 74 million tons production in 2014^14^. Oil extraction processes generate a considerable amount of canola meal, containing 35–40% w/w proteins^15^; there are limited uses of canola proteins in value-added products^16^, except as low-value animal and fish feed^17, 18^, protein supplements or substitutes in the bakery, dairy and egg products^19, 20^, and wood adhesive^15, 21^. Canola protein consists of two predominant storage proteins, cruciferin and napin at proportions of ~60% and ~20%, respectively. Cruciferin contains predominantly β-sheet-containing secondary structure (~75%) with a molecular weight of ~300–310 kDa^22, 23^. The β-sheet conformation tends to form the structure of superior mechanical strength such as spider and silkworm silks^24^ which inspired attempts to create a range of nanostructure by using β-sheet forming peptides, such as nanofibers^25^, twisted ribbon, belt, and fibril structures^26^. Therefore, it will be of great interest to explore β-sheet forming peptides in cruciferin.

Despite the successful fabrication of cruciferin-based nanoparticles in the previous study^27^, the fundamental question remains in regard to the mechanisms of particle assembly. In this previous study, after heat treatment at 120 °C, cruciferin were broken down into peptides. These observations, therefore, prompted us further investigate (1) what peptides exactly assembled the nanoparticles; (2) what were the main driving forces that contributed to this assembly; (3) whether the primarily β-sheet-containing cruciferin will generate peptides to form β-sheet-stabilized nanostructure. We hypothesized that peptides play a significant role in the cruciferin nanoparticle formation. Several studies have reported the nanoparticle formation after protein hydrolysis such as hybrid nanoparticles fabricated with fish scale gelatin hydrolysate^28^ and tannic acid, and peptidic nanoparticles prepared with whey hydrolysates^29^. However, the mechanism of particle formation as well as the responsible peptides for the particle formation have not been studied. Thus, the main objective of this study was to evaluate the feasibility of fabricating new cruciferin peptide-based nanoparticles using cruciferin derived peptides. We also aimed at elucidating the underlying mechanism of the particle development and identifying the sequence of peptides that were responsible for the nanoparticle formation.

## Results and Discussion

### Particle Characterisation

#### Nanoparticle Size, Polydispersity, and Turbidity

The optical properties and appearance of a colloidal dispersion usually reflect the size and quantity of the particles. To investigate the effects of peptide size on particle formation, several fractions of cruciferin peptides were obtained for fabricating particles. Among all fractions, cruciferin fraction < 10 kDa (F1) showed the greatest increase in turbidity after addition of Ca^2+^ (Table 1); the nanoparticles were deemed ‘formed’ when the suspension turbidity increased by at least 50% of the original dispersion, and also when no precipitate formed after adding calcium ions^30^. The average size of F1 formed nanoparticles (F1-NPs) was 82.12 nm, and 131.97 nm using 1.5 mM Ca^2+^ and 3 mM Ca^2+^, respectively, which is much smaller than cruciferin formed particles previously reported (175 ~ 255 nm)^27^. Thus, the F1 fraction might be an appropriate building block for fabrication of cruciferin-based nanoparticle.

**Table 1:** Effect of Ca^2+^ concentration on the suspension turbidity, particle size, and polydispersity (PDI) of cruciferin particles that were formed under different molecular weights.

Fractions	Ca^2+^ (mM)	Size (nm)	PDI	Increased turbidity (%)
<10 (kDa) (desalted)	0	104.07 ± 0.95^ab^	0.36 ± 0.01^bcd^	—
	1.5	82.12 ± 1.50^a^	0.20 ± 0.01^a^	106.05 ± 3.17^l^
	3	131.97 ± 12.20^abc^	0.36 ± 0.02^bcd^	98.51 ± 1.60^j^
10–30	0	956.00 ± 242.69^fg^	0.76 ± 0.07^i^	—
	1.5	167.87 ± 4.35^abc^	0.40 ± 0.02^def^	25.79 ± 0.54^c^
	3	270.93 ± 37.21^abc^	0.57 ± 0.08^gh^	103.54 ± 0.78^k^
30–50	0	826.50 ± 336.69^ef^	0.76 ± 0.15^i^	—
	1.5	138.43 ± 1.95^abc^	0.38 ± 0.01^cde^	7.30 ± 0.52^a^
	3	371.63 ± 9.39^bcd^	0.47 ± 0.01^ef^	85.15 ± 1.46^i^
50–100	0	1146.33 ± 114.42^g^	0.77 ± 0.05^i^	—
	1.5	1459.63 ± 400.54^h^	0.99 ± 0.02^h^	36.12 ± 0.39^e^
	3	1739.33 ± 368.04^i^	0.97 ± 0.06^jh^	54.29 ± 1.24^g^
100–300	0	753.93 ± 50.65^ef^	0.89 ± 0.02^j^	—
	1.5	608.47 ± 52.95^de^	0.61 ± 0.05^h^	42.37 ± 0.11 ^f^
	3	178.33 ± 1.03^abc^	0.22 ± 0.02^a^	22.29 ± 0.79^b^
>300	0	857.50 ± 79.10^ef^	0.79 ± 0.01^i^	—
	1.5	390.60 ± 20.92^cd^	0.49 ± 0.03^fg^	30.88 ± 0.14^d^
	3	166.47 ± 3.75^abc^	0.41 ± 0.04^def^	26.68 ± 0.39^c^
>10	0	396.50 ± 7.81^cd^	0.43 ± 0.01^def^	—
	1.5	307.63 ± 12.70^abc^	0.64 ± 0.11^h^	—
	3	145.07 ± 0.49^abc^	0.29 ± 0.02^abc^	29.42 ± 0.37^d^
Control	0	161.7 ± 2.08^abc^	0.28 ± 0.00^ab^	—
	1.5	185.50 ± 2.69^abc^	0.39 ± 0.02^de^	62.60 ± 0.21^h^
	3	143.60 ± 0.78^a^	0.39 ± 0.00^de^	62.79 ± 5.12^h^

Values within the same row that do not share a common superscript letter are significantly different (p < 0.05). Control was unfractionated sample.

**Figure 1:**
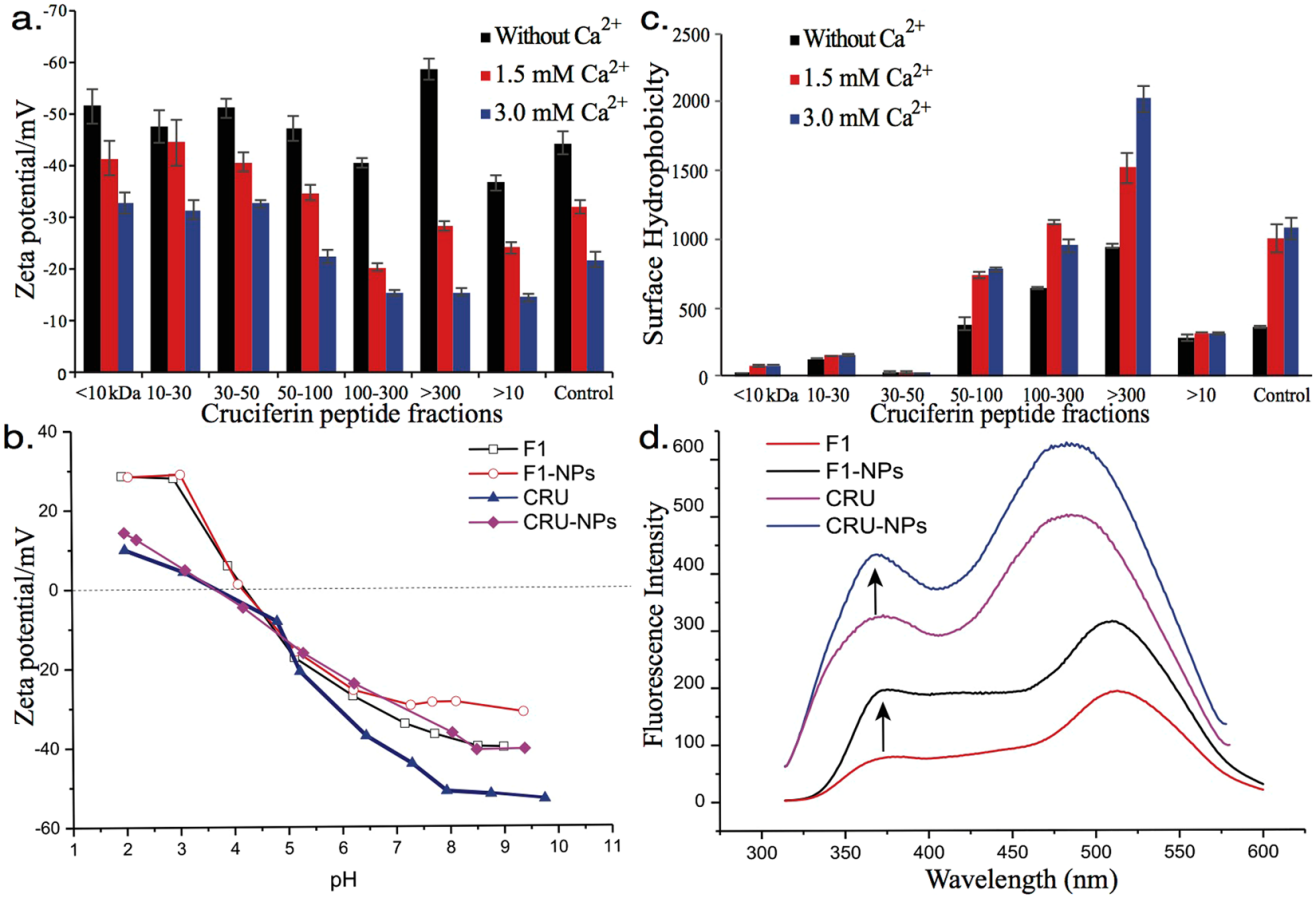
Changes in the (**a**) zeta potential and (**c**) surface hydrophobicity of cruciferin peptide fractions (Control was unfractionated sample). (**b**) Zeta potential of CRU (cruciferin peptides), F1 (cruciferin peptide fraction <10 kDa), CRU-NPs (nanoparticles formed from cruciferin peptides), and F1-NPs (nanoparticles formed from cruciferin peptide fraction <10 kDa) as a function of pH. (**d**) Intrinsic emission fluorescence spectra (excitation wavelength: 295 nm, slit: 10 nm) of CRU, F1, CRU-NPs, and F1-NPs.

Increasing Ca^2+^ concentrations in the F1 dispersion resulted in larger particle size and higher particle polydispersity (higher PDI). Akbari & Wu^27^ and Zhang *et al*.^30^ reported similar results for the effect of Ca^2+^ on the formation of nanoparticles from cruciferin and soy protein, respectively. They observed that the size of particles increased at increasing Ca^2+^ concentrations; particles precipitation might occur at higher Ca^2+^ concentration^27, 30^. The presence of high concentration of positively charged calcium ions could balance the negative charge of peptides, as indicated by the zeta potential (Fig. 1a), leading to precipitation or the formation of agglomerates due to decreased repulsion among particles^31^.

#### Zeta Potential Studies

To elucidate whether the surface charge was the major factor for the particle formation, zeta potential of the prepared nanoparticle dispersions was measured. The cruciferin peptide fractions exhibited zeta potential ranged from −36.8 mv to −58.67 mv (Fig. 1a); adding Ca^2+^ decreased the zeta potential (in terms of its absolute value), which indicated that adding Ca^2+^ reduced repulsive forces among peptides, contributing to the formation of nanoparticles^32^. Colloids with high zeta potential (negative or positive) indicate that particles are electrically stabilized; a low zeta potential tends to coagulate or flocculate^33^. In general, zeta potential below (±) 30 was considered as unstable for samples^34^. Thus, in the presence of 3.0 mM calcium ions, dispersion of peptide fractions of 100–300 kDa, >300 kDa, and >10 kDa exhibited the tendency to flocculate while nanoparticles formed by fractions <10 kDa were stable.

The pH point at which the zeta potential is zero is termed as the isoelectric point (*pI*)^35^. The *pI* of cruciferin peptide fraction <10 kDa (F1) and unfractionated cruciferin peptides (CRU) were approximately pH 3.6 and 4.2 (Fig. 1b), respectively. Particles at their isoelectric points were prone to agglomeration because electrostatic repulsion is not sufficient to overcome the attraction forces from dipole–dipole, hydrophobic, and van der-Waal’s interactions among the particles. There is no obvious difference between the isoelectric points (*pI*) of CRU-NPs and F1-NPs. However, the zeta potential of CRU-NPs and F1-NPs increased after particle formation at pHs (7.25 to 9.75) that were far from isoelectric points.

#### Surface Hydrophobicity (S_0_)

Surface hydrophobicity (*S*_0_) of cruciferin peptide increased at increasing molecular weights from 30 kDa to over 300 kDa with or without calcium (Fig. 1c). The *S*_*o*_ of fractions 100–300 kDa and >300 kDa were significantly higher than the unfractionated sample (control), while those of fractions <50 kDa were significantly lower than the control. This result suggested that the higher molecular weight fractions are more hydrophobic than the lower molecular weight fractions. Unlike the previous study where the surface hydrophobicity decreased^30^ after adding Ca^2+^, the *S*_*o*_ value increased in this study. This discrepancy is probably because the particles were mostly formed by hydrophobic peptides, which partly cover the surface of the particles. As indicated in a previous study, ANS could also bind to some proteins through electrostatic forces, in which ion pairs are formed between ANS sulfonate groups and cationic groups on the proteins^36^. Thus, adding Ca^2+^ into nanoparticle dispersion would enhance positive charge leading to more ANS binding to CRU-NPs and F1-NPs. Sarkar^37^ observed that ANS could move from its binding site in proteins to the silver nanoparticle surface. Thus, a similar movement of ANS may also have occurred after fabrication of CRU-NPs and F1-NPs.

#### Intrinsic Emission Fluorescence Spectra

Tryptophan is the primarily fluorescent residue in the protein structure that can be selectively excited at 295 nm which can avoid excitation of tyrosine^38^. Because the tryptophan fluorescence is strongly influenced by its local microenvironment^39^, it is applicable to study the conformational changes of proteins during the assembly of cruciferin nanoparticle by intrinsic fluorescence analysis of tryptophan. All intrinsic emission fluorescence spectra exhibited dual peaks (Fig. 1d); peaks at around 500 nm were probably derived from the pigment in canola^40, 41^. The intensity of fluorescence spectrum increased after particle formation, which is in agreement with our previous study^27^. Increased intrinsic fluorescence intensity suggested that the polarity of microenvironment surrounding tryptophan changed to “less-polar”^42^, indicating peptide assembly. Blue shifts from 385 to 378 nm and from 373 to 369 nm were also observed after particle formation for F1-NPs and CRU-NPs, respectively, indicated the increased hydrophobicity around tryptophan residues.

#### Secondary Structure

**Figure 2:**
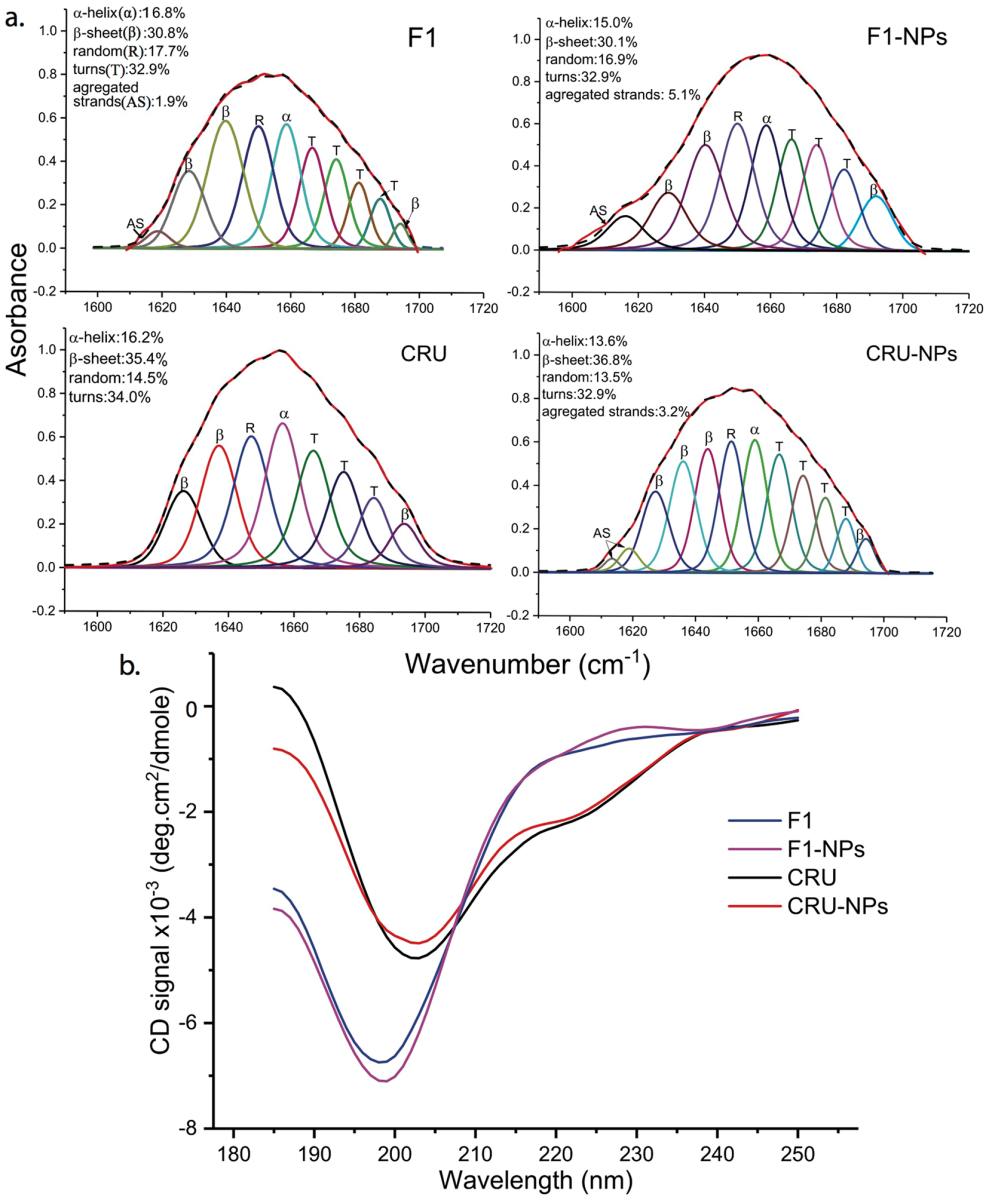
Fourier self-deconvoluted absorbance spectra (**a**) and circular dichroism spectra (**b**) of CRU (cruciferin peptides), F1 (cruciferin peptide fraction < 10 kDa), CRU-NPs (nanoparticles formed from cruciferin peptides), and F1-NPs (nanoparticles formed from cruciferin peptide fraction < 10 kDa) at amide I band between 1,600–1,700 cm^−1^. The dotted lines resulted from self-deconvolution of experimental data (outside solid line) and inside solid lines are fitted curves.

The FTIR spectrum, especially in the range of 1,600–1,700 cm^−1^, is a reliable indicator of secondary structures of proteins and peptides^43^. The lyophilized cruciferin samples (F1, F1-NPs, CRU, and CRU-NPs) were examined by attenuated total reflectance (ATR) FTIR spectroscopy (Fig. 2a), which included an expansion of the amide I region for secondary structure analysis. The amide I band was transformed to yield a fitted, self-deconvoluted set of bands, from which the secondary structural elements were determined. The outside solid line (after deconvolution) and the dotted line (before deconvolution) fit well, which indicated an ideal peak fitting result. CRU-NPs and F1-NPs adopted 69.7% and 63.0% β-sheets and turns, respectively, which was consistent with our hypothesis that the primarily β-sheet-containing cruciferin was likely to generate peptides to form β-sheet-stabilized nanostructure.

Both F1-NPs and CRU-NPs showed less α helix while presented more aggregated strands than F1 and CRU. The assignment of aggregated strands between 1,610 and 1,625 cm^−1^ was according to Jackson & Mantsch^44^, and the enhanced aggregated strands were owing to the increased numbers of hydrophobic peptides in the particles^45^, suggesting that a very compact structure of F1-NPs and CRU-NPs was formed after adding Ca^2+^.

The circular dichroism (CD) spectra (Fig. 2b) of cruciferin and F1 before and after particle formation were measured to confirm the changes in structural characteristics and complement the FTIR result. All CD spectra in the far-UV region exhibited significant negative ellipticities from 190 to 250 nm, which was owing to the heat treatment of both CRU and F1. The F1 and F1-NP show classical random coils and other two helical conformations (week peaks at 222 nm). Secondary structures were predicted by CAPITO^46^; random coil, β-sheet, and α-helix were 59%, 40% and 1% for F1, 60%, 38% and 2% for F1-NPs, 58%, 41, 1% for CRU, 51%, 44% and 5% for CRU-NPs, respectively. It should be noted, in comparison to FTIR, that CD was reported to be less reliable for the study of aggregated proteins as a result of interference from light scattering by aggregates^47, 48^.

#### Particle Morphology

**Figure 3:**
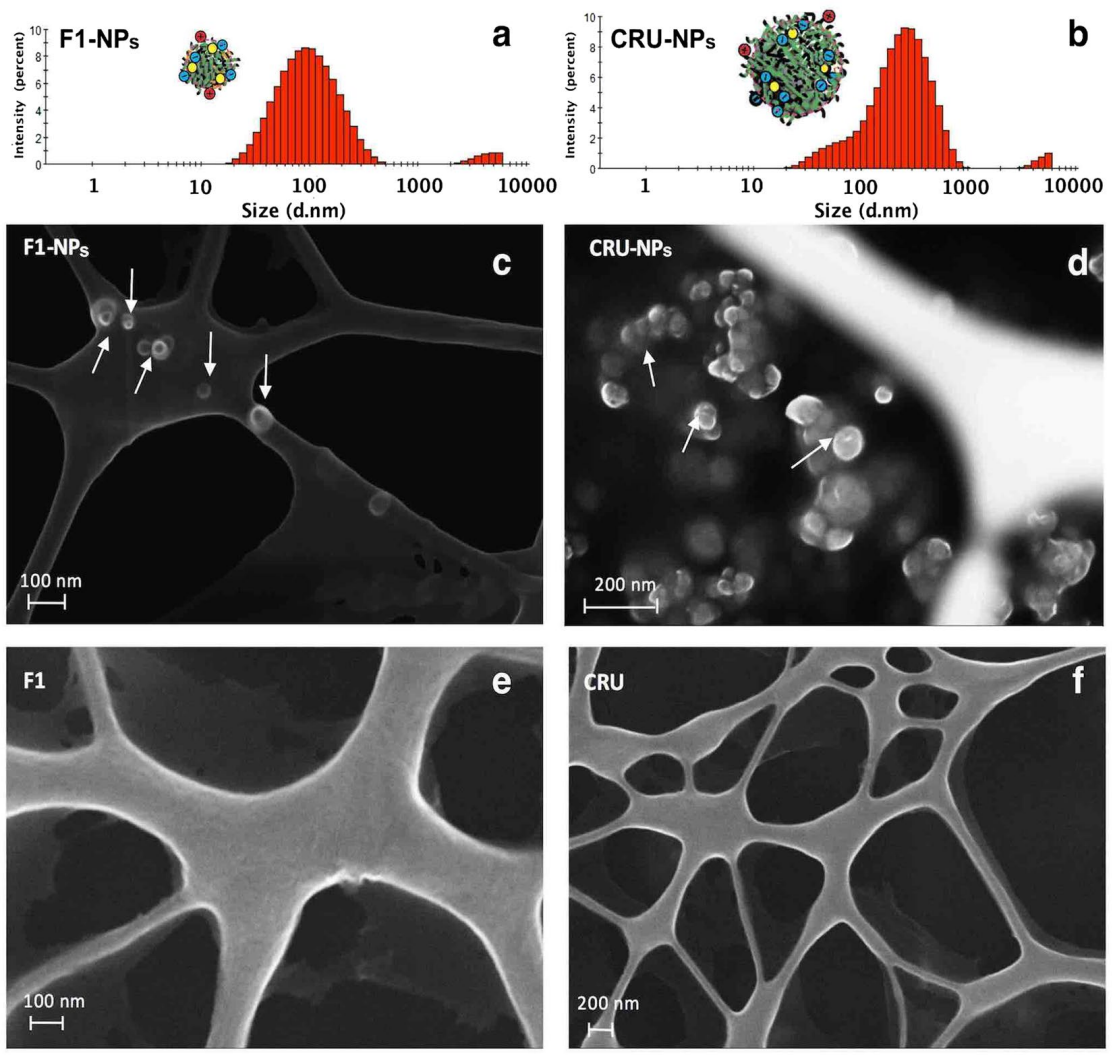
Intensity-based size distribution histograms of (**a**) F1-NPs (nanoparticles formed from cruciferin peptide fraction <10 kDa) and (**b**) CRU-NPs (nanoparticles formed from cruciferin peptides) from the dynamic light scattering and SEM micrographs for representative (**c**) F1-NPs, (**d**) CRU-NPs, (**e**) F1 (cruciferin peptide fraction <10 kDa), and (**f**) CRU (cruciferin peptides) on copper grids.

Intensity-based measurement of hydrodynamic diameters showed that the z-average size of CRU-NPs was 185 nm (Fig. 3a **)**, which was greater than the F1-NPs with a z-average size of 82 nm (Fig. 3b), suggesting that smaller cruciferin peptides were more likely to form smaller nanoparticles.

To further confirm the mean diameter and spherical morphology of F1-NPs and CRU-NPs, SEM was used to image the morphology of the particles. Many studies used aluminum pans or silicon discs for nanoparticle deposition^32, 49^. However, because most proteins also show spheral structures in their dry states, the results will be confounded by whether the spheral structure came from the fabricated particle or from proteins or peptides. For example, as shown in the SEM results by Teng *et al*.^32^, soy protein isolate showed a spheral structure. In the present study, copper grids with carbon film were used to deposit samples. This grid held the particles, but it allowed the extra liquid to pass through the holes in the grid. The SEM micrographs of both F1-NPs and CRU-NPs showed that they exhibited spherical structures (Fig. 3c,d). Velichko *et al*.^50^ demonstrated that this type of spherical structures was held by hydrophobic interaction rather than hydrogen bonding in a molecular simulation study. The SEM micrographs confirmed that the sizes of CRU-NPs and F1-NPs were slightly smaller than those in the DLS results because NPs dehydrated under SEM observation. The cruciferin peptides (Fig. 3e) and F1 (Fig. 3f) before particle fabrication did not show particles on the copper grid.

#### Driving Force Analysis: Effect of Dissociating Reagents

**Figure 4:**
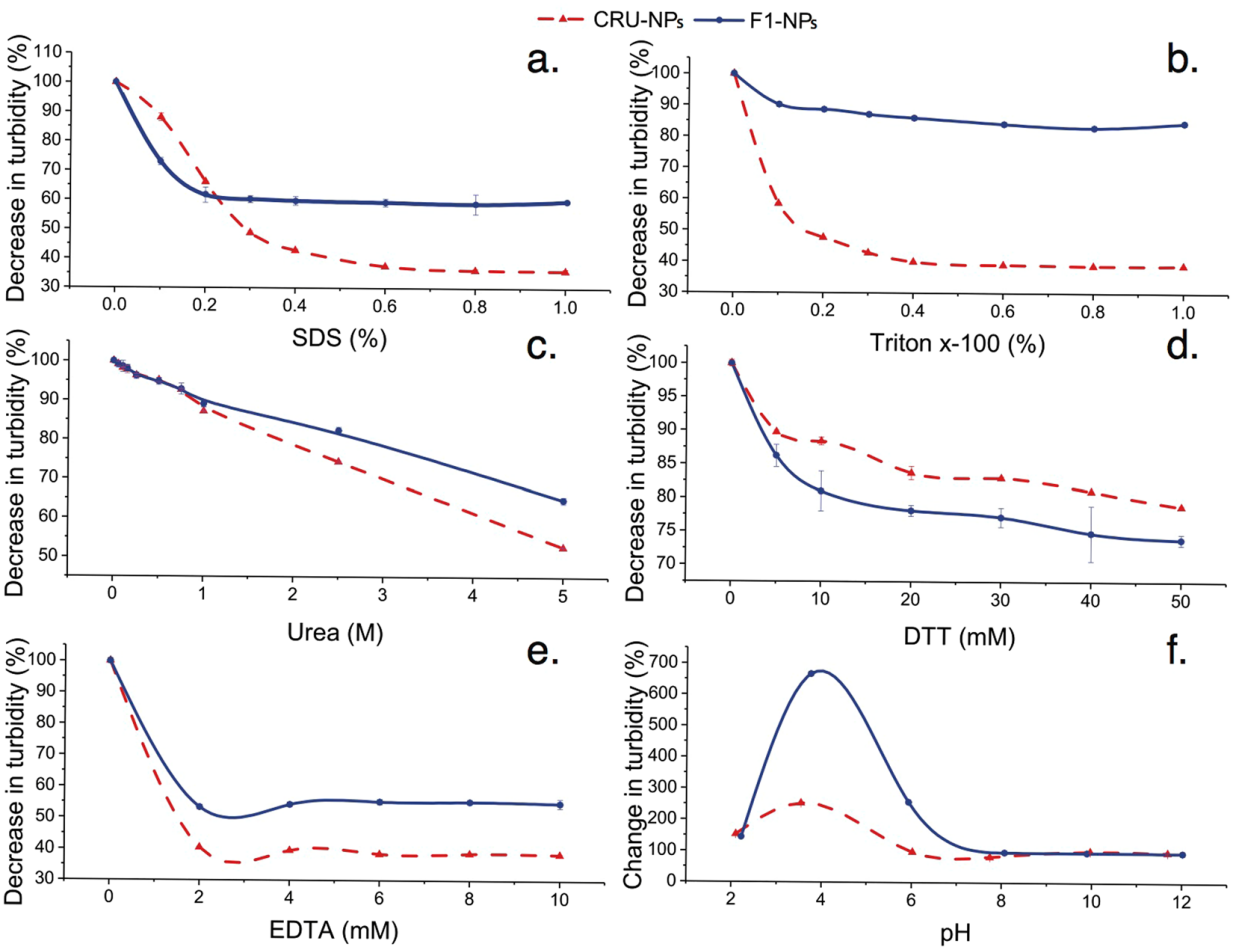
Decrease in turbidity of F1-NPs (nanoparticles formed from cruciferin peptide fraction <10 kDa) and CRU-NPs (nanoparticles formed from cruciferin peptides) in the presence of (**a**) SDS, (**b**) Triton x-100, (**c**) urea, (**d**)1,4-dithiothreitol (DTT), (**e**) ethylenediaminetetraacetic acid (EDTA) and changes in turbidity at (**f**) various pH values.

Stability is one of the critical aspects ensuring the integrity and function of nanoparticles. The ability to assemble stable nanostructures from peptides is due to structural complementarities that are coupled with hydrogen bonding, electrostatic interaction, and hydrophobic affinity^51^. These molecular forces tend to be disrupted by most dissociating reagents. To elucidate the driven force responsible for the formation of CRU-NPs and F1-NPs, five dissociating reagents (SDS, Triton x-100, EDTA, urea, and DTT), and various pHs, were used to determine the stability of CRU-NPs and F1-NPs. Changes in the turbidity of particle dispersion were used to determine the stability of the nanoparticles after the addition of dissociating reagents. The turbidity of the nanoparticle dispersions all decreased at increasing concentrations of dissociating reagents (Fig. 4).

SDS is an anionic surfactant that weakens hydrophobic interactions and masks the intrinsic charges in proteins. The addition of SDS to the dispersions of CRU-NPs and F1-NPs resulted in nanoparticle disassociation, as evidenced by a gradual decrease in the turbidity at increasing SDS concentrations (Fig. 4a). The CRU-NPs were more susceptible to SDS than F1-NPs at SDS concentrations higher than 0.3%. The turbidity of CRU-NPs decreased to 40% of initial turbidity, but F1-NPs retained as much as 60% of their original turbidity. This difference indicated that F1-NPs might have a more compact structure than CRU-NPs.

Triton X-100 is a nonionic surfactant that has a hydrophobic group and a hydrophilic polyethylene oxide chain. The main effect of Triton X-100 is to associate with the hydrophobic parts of protein, thereby conferring solubility to protein. The CRU-NPs were disrupted by Triton X-100, as evidenced by the dramatic decrease in the turbidity of CRU-NPs dispersion. However, F1-NPs were much more stable compared with their SDS-treated counterpart, which indicated that the formation of F1-NPs was directed mostly by the electrostatic interaction than the hydrophobic interaction because Triton X-100 did not mask the charges in particles. Thus, the peptides responsible for CRU-NPs were more hydrophobic than those for F1-NPs.

Urea can disrupt proteins by disturbing the hydrophobic interaction and by binding to the amide units via hydrogen bonds and competing with existing inter- and intramolecular hydrogen bonds which stabilize protein structures^52^. Urea significantly decreased the turbidity of the dispersion of CRU-NPs and F1-NPs, which suggested that both hydrophobic interactions and hydrogen bonds contributed to the internal structure of CRU-NPs and F1-NPs.

DTT reduced the turbidity of F1-NPs only to 75% of its original turbidity. CRU-NPs and F1-NPs were much more stable under DTT treatment compared to the other four disrupting agents. DTT is a reducing agent, which is known to disrupt disulfide bonds. Thus, the disulfide bonds were not the main strength to maintain the structure of CRU-NPs and F1-NPs.

The addition of EDTA, a calcium chelating agent, had a significant effect on the turbidity of both dispersions. The turbidity of CRU-NPs and F1-NPs decreased to 40% and 50% of their initial turbidity, respectively, and then remained constant at increasing EDTA concentrations. The results indicated that calcium was incorporated in the particles and facilitated the formation of both CRU-NPs and F1-NPs through electrostatic interactions.

The turbidity of CRU-NPs and F1-NPs was unchanged at the pH range of 8–12. This observation agreed with the results of zeta potential showing that the electric repulsion among particles was high enough to maintain the stability of the particle dispersion. Starting from pH 6.5, there was a sharp increase in the turbidity at decreasing pHs and reached the maximum at pH 4. This sharp increase was owing to enhanced attraction among particles, which might induce the flocculation of particles. When pH was decreased to less than 4, the turbidity began to decrease, which was a result of the disassociation of particle flocculation at extreme acidity.

### Peptide Identification

#### SDS-PAGE

**Figure 5:**
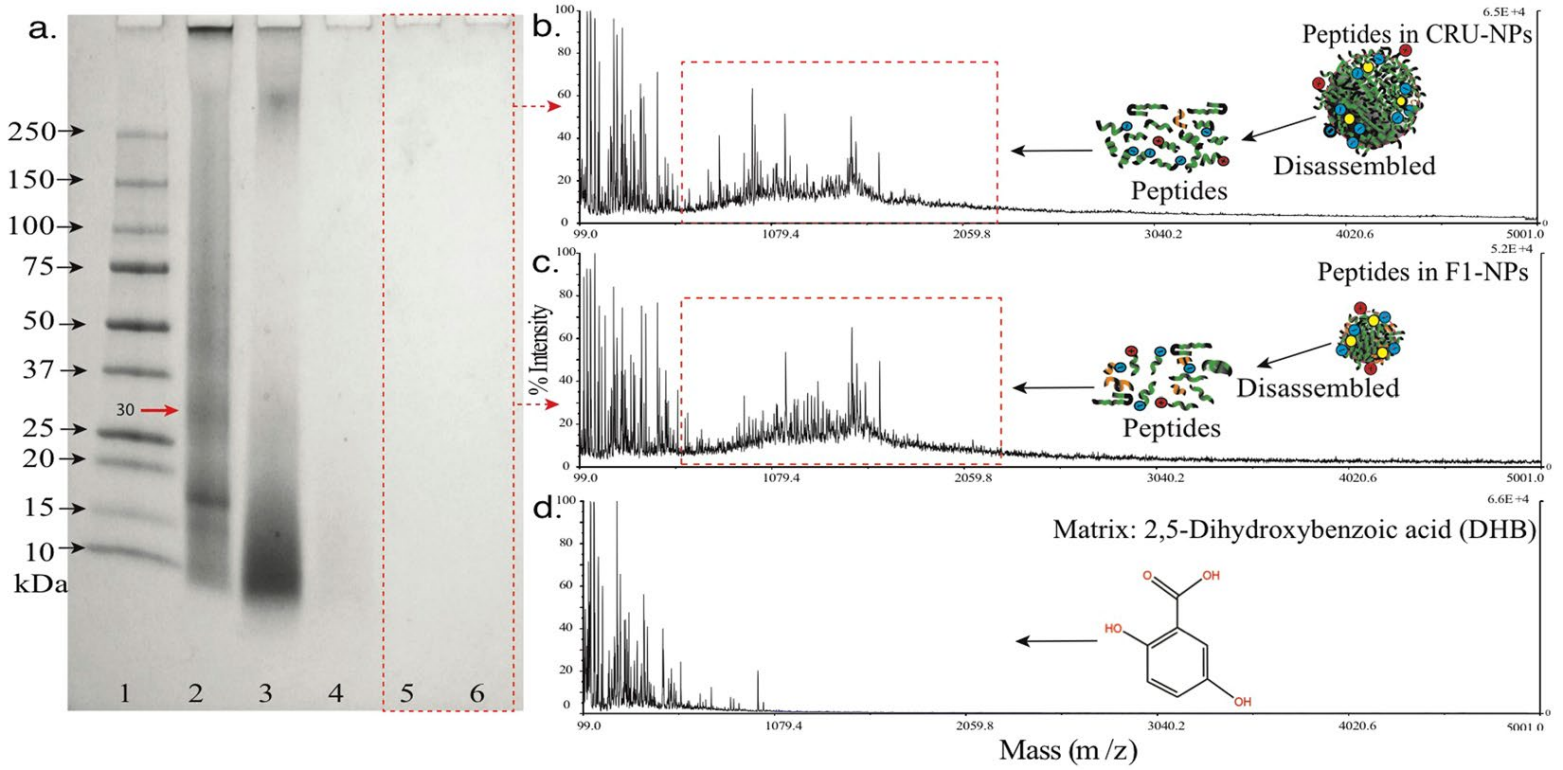
(**a**) SDS-PAGE of cruciferin samples in the presence of 5% ME (lanes 1, marker; lane 2, cruciferin (control); lane 3, CRU (cruciferin peptides); lane 4, F1 (cruciferin peptide fraction <10 kDa); lane 5, CRU-NPs (nanoparticles formed from cruciferin peptides); lane 6, F1-NPs (nanoparticles formed from cruciferin fraction <10 kDa)). Matrix-assisted laser desorption/ionization time-of-flight (MALDI-TOF) mass spectrometry of peptides disassembled from (**b**) F1-NPs, (**c**) CRU-NPs, and (**d**) matrix, 2,5-Dihydroxybenzoic acid (DHB).

The SDS-PAGE was conducted to evaluate the molecular weight of peptides that are responsible for the assembly of CRU-NPs and F1-NPs (Fig. 5a). Lane 2 shows two major bands with estimated molecular weights of 18 and 30 kDa in unheated cruciferin sample. Several cruciferin bands were disappeared because of high pH treatment^27^. Lane 3 represents the heated cruciferin showing a broad range of the bands at around 10 kDa, which indicated that cruciferin was broken down into mixed peptides after the heating treatment at pH 12. The CRU-NPs and F1-NPs were separated from the dispersion by high-speed centrifugation (16,000 g for 30 min). The particles were then disassociated into peptides in the presence of SDS. Lanes 4 and 5 exhibit the polypeptides fabricating CRU-NPs and F1-NPs, respectively. These two lanes did not show clear bands, which suggested that the molecular weight of the peptides that were responsible for forming CRU-NPs and F1-NPs were smaller than 10 kDa.

#### Matrix-Assisted Laser Desorption/Ionization Time-of-Flight Mass Spectrometry (MALDI-TOF-MS)

Since no band showed in SDS-PAGE, the molecular weight of CRU-NPs and F1-NPs peptides was further studied by MALDI-TOF-MS. As shown in Fig. 5b,c, CRU-NPs and F1-NPs were composed of a mixture of peptides below 3 kDa, which was in agreement with the SDS-PAGE result. The primary molecular weight of CRU-NPs and F1-NPs peptides ranged from 500 to 2000 Da, indicating that small peptides were responsible for CRU-NPs and F1-NPs formation. Small peptide-based nanoparticles were also reported by Collins *et al*.^53^ who assembled nanoparticles for delivery of hydrophilic moieties to the cytosol by using peptides with 20 amino acid residues. Doll *et al*.^54^ designed a peptide nanoparticle with a minimal chain length of 36 amino acids. However, these peptide nanoparticles were fabricated using synthetic peptides, which might exhibit potential toxicity or less biocompatibility though they were well-defined and fine-tuneable.

#### Liquid Chromatography−Tandem Mass Spectrometry (LC−MS/MS)

**Figure 6:**
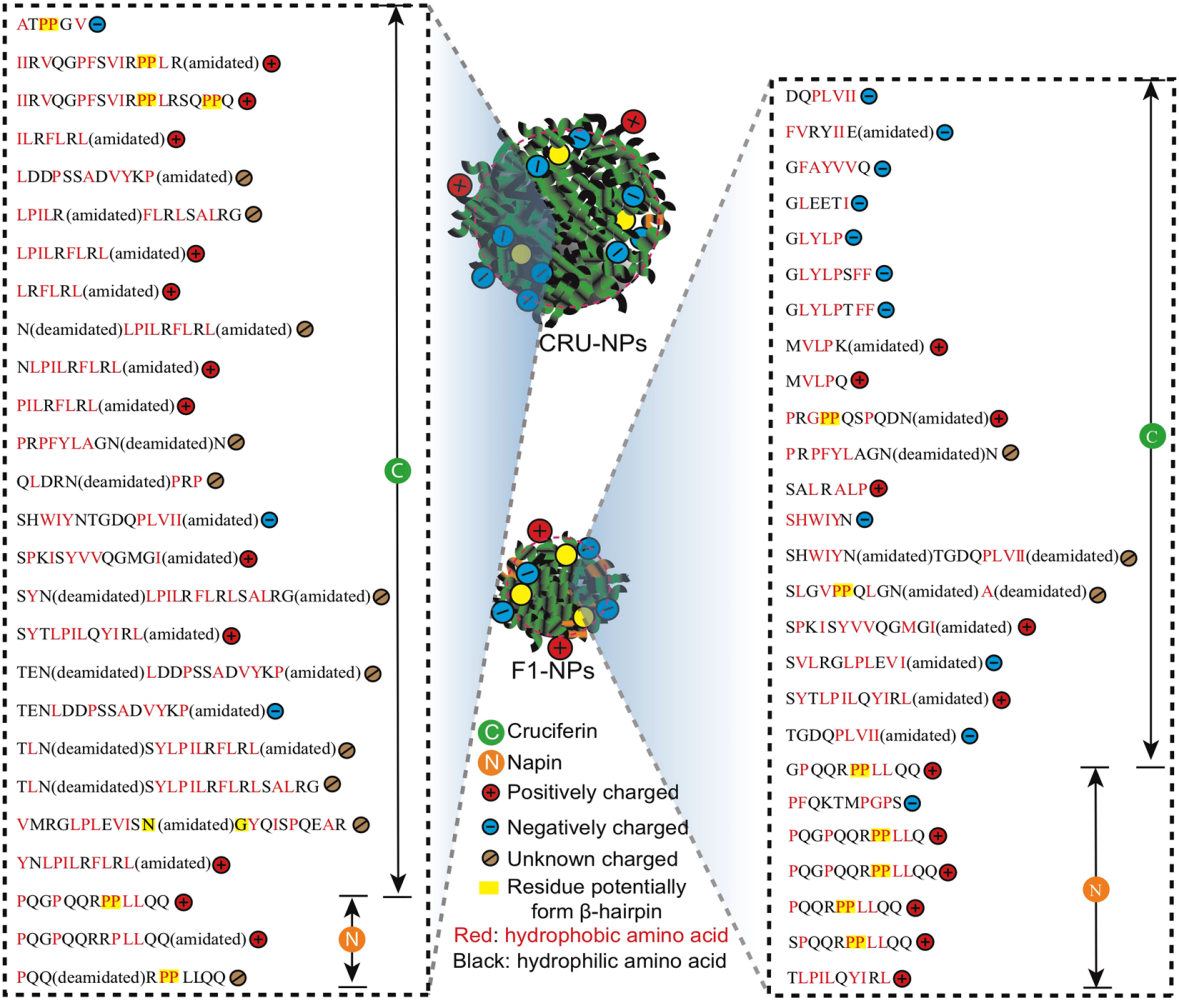
Peptides responsible for the formation of CRU-NPs (nanoparticles formed from cruciferin peptides) and F1-NPs (nanoparticles formed from cruciferin fraction < 10 kDa). The charge at pH 9 was calculated based on http://pepcalc.com/. The charge was labeled as unknown for the peptides with unenclosed modifications on this website.

To better understand the peptides responsible for CRU-NPs and F1-NPs, the peptides from CRU-NPs and F1-NPs were sequenced by LC-MS/MS. Twenty-six peptides were identified from F1-NPs (Fig. 6), which were mainly derived from cruciferin. Consistent with our observations on zeta potential in which all mixed peptides had negative charges at pH 9, eleven of the identified peptides had negative charges at pH 9. The electrical charges of three peptides were unknown because the modification in their amino acids altered their isoelectric points. These negative charges indicated that the formation of the particles was led partially by the electrostatic attraction between the negatively charged side chain of peptides and calcium ions. Also, when the negative charges were neutralized by Ca^2+^, it facilitated hydrophobic interaction to form the particles. In addition, the length of peptides responsible for F1-NPs ranged from 5 to 15 amino acids which were consistent with the MALDI-TOF-MS results (Fig. 5c).

Twenty-six peptides were identified from CRU-NPs (Fig. 6). Most of the identified peptides were also derived from cruciferin, with peptide length ranged from 5 to 22. The average peptide length in CRU-NPs was higher than that in F1-NPs. The peptides responsible for the formation of CRU-NPs were more hydrophobic than the peptides in F1-NPs, which agreed with the previous results of the stability change in both particles after addition of SDS and Triton x-100. Only three identified peptides had a negative charge at pH 9, suggested that the assembly of peptides into particles was largely directed by hydrophobic interaction other than electrostatic interaction between peptides and calcium ions. The sequences identified further supported our discussion that the increased surface hydrophobicity after particle formation was due to the proximity of hydrophobic peptides after Ca^2+^ addition. The hydrophobic peptides identified also explained the high encapsulation efficiency and loading capacity of hydrophobic β-carotene in Cruciferin/Ca particles reported by Ali and Wu^27^.

Both CRU-NPs and F1-NPs peptides consisted of a significant amount of proline (P) and glycine (G), both of which are commonly found in turns such as β-hairpins or in the edge strands of a β-sheet^55^. This observation agreed with the high proportions of turns content (over 30%) in CRU-NPs and F1-NPs determined by FTIR. The unique cyclic structure of proline and the shortest side chain of glycine made them ideally suited for beta turn^55^. Inspired by such structures, proline and glycine were incorporated to make hairpin peptides (such as (VK)_4_-VDPPT-(KV)_4_–NH_2_, CVLKNGEWHC-NH_2_), which can be applied for drug release and cell culture^56, 57^. As residues PP and NG were extensively demonstrated to be the turn sequence of hairpin conformation^58, 59^, there were 6 and 7 β-hairpin peptides being detected in CRU-NPs and F1-NPs, respectively (see peptides with a yellow background in Fig. 6), which may contribute to the spherical structures of CRU-NPs and F1-NPs.

**Figure 7:**
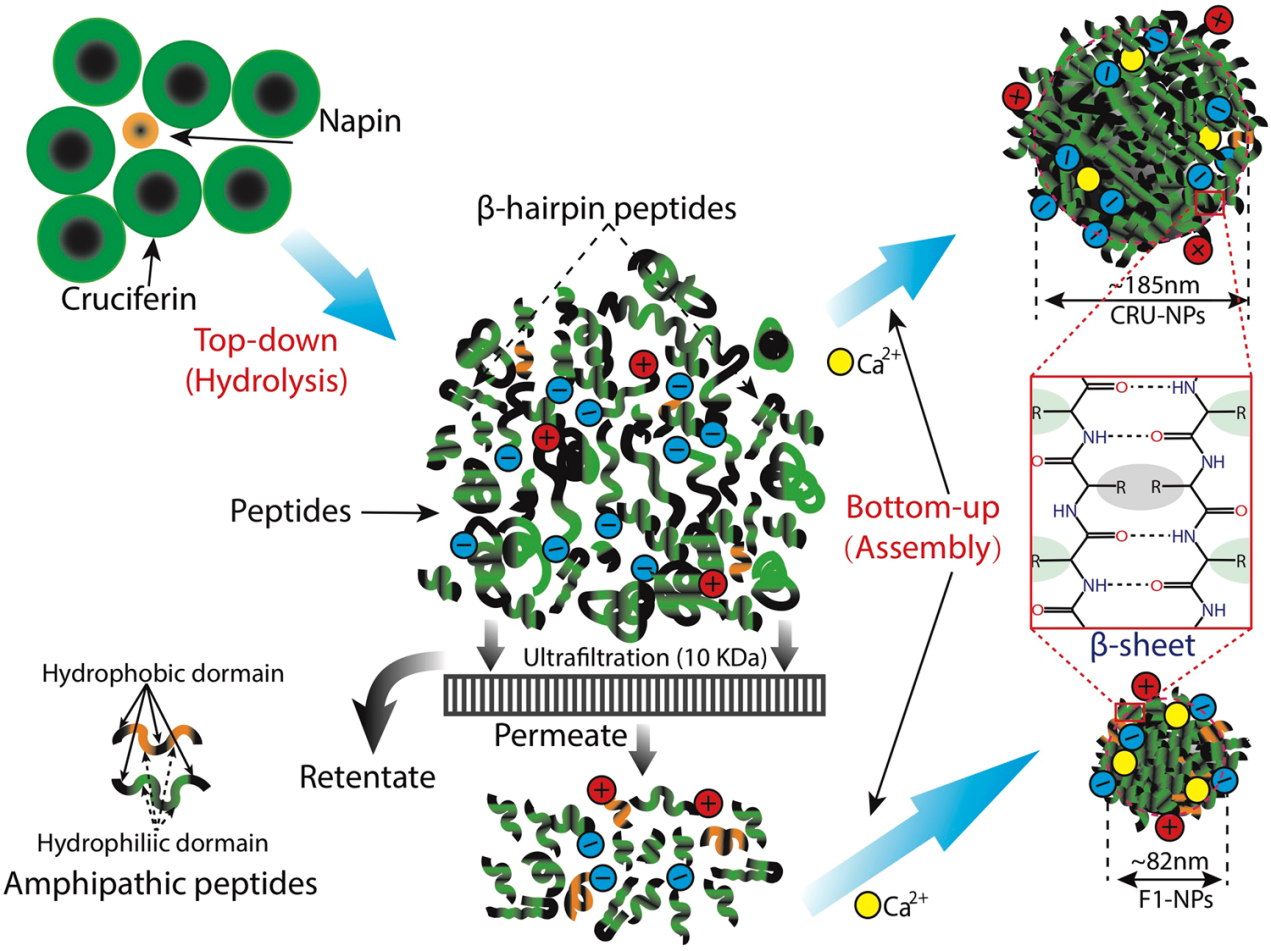
Schematic illustration of the assembly of cruciferin nanoparticles.

As shown in Fig. 6, it is interesting to note that both CRU-NPs and F1-NPs were fabricated with amphipathic peptides, composed of alternating hydrophobic (X) and hydrophilic (Z) residues and shared a general pattern of X_n1_Z_n2_X_n3_Z_n4_ (n_1–4_ = 0~5) motif. The amphipathic peptides favoured forming β-sheet structure^60^, which was consistent with our FTIR result that β-sheet and turns dominated the secondary structure in CRU-NPs and F1-NPs. Thus, based on the above observations, the mechanism of the assembly of cruciferin nanoparticles could be illustrated as shown in Fig. 7. Our study showed for the first time that proteins were broken down into peptides at high temperature and high pH and then the particles were formed by peptide assembly rather than gelation.

## Conclusions

Our study elucidated for the first time, using cruciferin as an example, that it is the small amphipathic peptides, with 5–22 amino acid residues in a general pattern of X_n1_Z_n2_X_n3_Z_n4_ (n_1–4_ = 0~5), responsible for the particle formation. These amphipathic peptides primarily adopted β-sheet and turn conformations; electrostatic interactions and hydrophobic interactions were the two main forces responsible for the particle formation.

## Materials and Methods

### Materials

Defatted canola meal, obtained from Richardson Oilseed Company (Lethbridge, AB, Canada), was ground, passed through a 35-mesh screen and stored at −20 °C for further use. The precast 4–20% gradient gels and the molecular weight markers were both purchased from Bio-Rad Laboratories Inc. (Bio-Rad Laboratories Inc., Hercules, CA, USA). 1-anilino-8-naphtalene-sulphonate (ANS), SDS, Triton x-100, urea, and 1,4-dithiothreitol (DTT) were purchased from Sigma (Sigma, St. Louis, MO, USA). Ethylenediaminetetraacetic acid (EDTA) was obtained from Thermo Fisher (Thermo Fisher Scientific, Inc., Waltham, MA).

### Alkali Hydrolysis of Cruciferin

Cruciferin was extracted through acidic washing (pH 4), alkaline extraction (pH 12.5), isoelectric precipitation (pH 4), and ultrafiltration according to Akbari and Wu^61^. Cruciferin was dissolved in deionized water at a concentration of 10 mg/mL, and the pH was adjusted to 12. The dispersion was then stirred for 1 hour at 500 rpm and 30 mL was transferred to capped glass screw tubes. The cruciferin was then alkali hydrolysed by incubating the tubes at 120 °C in canola oil for 30 min as previously reported^27^. The heated sample was cooled down to room temperature and then freeze-dried for further use.

### Fractionation of Cruciferin Peptides by Ultrafiltration

The resulting cruciferin alkali hydrolysate was further ultrafiltrated using 10, 30, 50, 100, and 300 kDa molecular weight cut-off (MWCO) membranes (Spiral-wound ultrafiltration system, Millipore, Bedford, MA, US). The hydrolysate was fractionated using a 10 kDa MWCO membrane; the 10 kDa retentate was then subjected to ultrafiltration over the 30 kDa MWCO membrane, and the resultant retentate and permeate were collected. The same process was repeated for 50, 100, and 300 kDa MWCO membranes. The second set of experiments was conducted individually by ultrafiltrating the protein hydrolysate over the 10 kDa MWCO membrane. All permeates and retentates were collected and freeze-dried for further analysis^62^.

### Preparation of Cruciferin Nanoparticles

Nanoparticles were fabricated from cruciferin peptides and its fractions using the method developed by Akbari and Wu^27^ with some modifications. In brief, cruciferin peptides and its fractions were dissolved in deionized water at a concentration of 6 mg/mL, followed by the removal of large aggregates by centrifugation at 10,000 g for 15 min. The pHs of the above cruciferin solution were adjusted to 9. To initiate the formation of nanoparticles, CaCl_2_ (9 mM and 18 mM) was added dropwise to the dispersion under continuous stirring (500 rpm) to induce particle formation with a final concentration of CaCl_2_ of 1.5 mM and 3 mM in the protein solutions. The particles were further characterised.

### Turbidity

The turbidity of the formed particle suspensions was measured using a UV–Visible spectrophotometer (Evolution 60 S, Thermo Scientific, MA, USA) at 600 nm. The percentage of measured values compared to the original turbidity value was expressed as relative turbidity.

### Particle Size and Zeta Potential Measurements

The mean particle size, polydispersity index (PDI), and zeta potential of the nanoparticle were determined by dynamic light scattering (DLS) using Malvern Nanosizer ZS (Malvern Inc., Malvern, UK) at 25 °C. The DTS1070 folded capillary cells were used for measurements of both size and zeta potential. The samples were prepared and measured in triplicate. The cells were filled slowly to avoid air bubbles.

### Surface Hydrophobicity (*S*_0_)

Surface hydrophobicity was determined with the anionic fluorescence probe of 1-anilino-8-naphtalene-sulphonate (ANS), using the method developed by Alizadeh-Pasdar *et al*.^63^ with slight modifications. Briefly, the samples were diluted to five concentrations that ranged from 0.006% to 0.030% using 0.1 M phosphate buffers at pH 9. A 20 µL aliquot of 8 mM ANS that was prepared in the same buffer was added to 4 mL of the diluted protein solution and incubated for 10 min in dark. ANS fluorescence intensity was measured at the emission wavelength of 470 nm and at 390 nm excitation wavelength with slit widths of 5 nm using a Shimadzu RF-5301PC spectrofluorophotometer (Shimadzu Corp., Kyoto, Japan). The slope of the linear regression of net fluorescence values vs. protein concentrations (mg/mL) was used as *S*_0_.

### Intrinsic Emission Fluorescence Spectra

The intrinsic fluorescence emissions of the samples were measured using a Shimadzu RF-5301PC spectrofluorophotometer. The excitation wavelength was set at 295 nm and emission was scanned from 300 to 600 nm. The excitation and emission slit width were set at 10 nm. All samples were measured at 10 mg/ml in 10 mM phosphate buffer (pH 9.0).

### Attenuated Total Reflection Fourier Transform Infrared (ATR-FTIR) Spectroscopy

For ATR-FTIR, the freeze-dried samples were further dried in a vacuum desiccator with phosphorous pentoxide for 48 h. ATR-FTIR spectra of the milled samples were recorded in the range of 4,000–400 cm^−1^ using a Thermo Fisher Scientific Nicolet iS50 FTIR Spectrometer (Madison, WI, USA). The recorded spectra were self-deconvoluted in the amide I band region (1,700–1,600 cm^−1^) using Omnic 8.1 software at a bandwidth of 25 cm^−1^ and an enhancement factor of 2.5^27^. The peak fitting was performed using Gaussian components. The fitted amide I bands were assigned to protein secondary structures according to wavenumber ranges that were established previously^43, 44, 64^.

### Circular Dichroism (CD)

The far-UV CD spectra of the cruciferin samples (1 mg/mL) in 10 mM phosphate buffer at pH 9 were measured using Olis DSM 17 CD spectrophotometer (Bogart, GA, USA). The path length of the quartz cell was 0.2 mm, and the spectrum represented an average of five scans that were collected in 1 nm step at a rate of 20 nm/min over the wavelength range of 190–250 nm. The raw data were corrected by conversion to mean residue ellipticity. The CAPITO method was used to perform the CD curve fitting and quantify the secondary structure^46^.

### Scanning electron microscope (SEM)

Morphology of the prepared particles was visualized using a scanning electron microscopy (SEM, Zeiss Sigma FESEM). The particles were freshly prepared, and a drop was placed on a copper grid with carbon film. The extra liquid was absorbed with tissue paper at the edge of the grid, and then air-dried. The grid then adhered to a specimen stub with conductive carbon tapes.

### SDS-PAGE

SDS-PAGE was carried out as described by Uruakpa and Arntfield^65^. Cruciferin samples (2 mg/ml) were diluted to a ratio of 1:1 with Laemmli sample buffer with 5% β-mercaptoethanol and then heated to 85 °C for 10 min. An aliquot of each sample (15 µL) and molecular weight markers (10 µL) were loaded on precast Mini-Protean gels (4–20% gradient gels). To visualize the protein bands, the gel was stained with Coomassie brilliant blue R250 and destained using a solution composed of 50%, 40%, and 10% (V/V) of distilled water, methanol, and acetic acid, respectively. The gel was scanned in an Alpha Innotech gel scanner (Alpha Innotech Corp., San Leandro, CA, USA).

### MALDI-TOF-MS

The F1-NPs (nanoparticles formed from cruciferin peptide fraction < 10 kDa) and CRU-NPs (nanoparticles formed from whole cruciferin peptides) were collected from the dispersion by centrifugation at 16,000 g for 30 min and were washed twice with distilled water. The particles were then disassociated by EDTA (2 mM) and urea (5 M). The resultant solutions were desalted by an Oasis HLB 1 cc Vac Cartridge (Waters, Milford, MA, USA). The desalted peptides were mixed at a ratio of 1:1 with 20 mg/ml 2,5-Dihydroxybenzoic acid (DHB) in 50% acetonitrile/H_2_O, and 0.1% trifluoroacetic acid. An aliquot (1 μl) mixture was dropped onto the metal target plate and air-dried at room temperature. Spectra were recorded using a Perspective Biosystems Voyager Elite MALDI-TOF mass spectrometer. Four independent spectra were collected with over 500 shots at random positions on the dry spots of the mixture of sample.

### LC-MS/MS

The desalted sample from MALDI-TOF-MS analysis was also subjected to LC–MS/MS analysis using Waters ACQUITY UPLC system connected online Waters (Micromass) Q-TOF Premier (Milford, MA, USA) following the method described by Shen *et al*.^66^. An MS/MS full scan was performed for each sample with an m/z range of 100–1,990 Da. The peptide sequences were identified from the monoisotopic mass. The Auto De Novo, Peaks DB Search, and Spider of Peaks 8.0 software (Bioinformatics Solutions Inc., Waterloo, ON, Canada) in combination with Mascot MS/MS search were used to process the MS/MS data.

### Statistical Analysis

Statistical analysis was performed with the IBM SPSS Statistics 20 (IBM Corporation, NY, USA) using one-way ANOVA. Duncan’s multiple range tests were used to test for difference between means (significance was defined at *p *< 0.05).

### Data Availability

All data generated or analysed during this study are included in this published article.
